# Systematic review of mental health symptom changes by sex or gender in early-COVID-19 compared to pre-pandemic

**DOI:** 10.1038/s41598-022-14746-1

**Published:** 2022-07-06

**Authors:** Tiffany Dal Santo, Ying Sun, Yin Wu, Chen He, Yutong Wang, Xiaowen Jiang, Kexin Li, Olivia Bonardi, Ankur Krishnan, Jill T. Boruff, Danielle B. Rice, Sarah Markham, Brooke Levis, Marleine Azar, Dipika Neupane, Amina Tasleem, Anneke Yao, Ian Thombs-Vite, Branka Agic, Christine Fahim, Michael S. Martin, Sanjeev Sockalingam, Gustavo Turecki, Andrea Benedetti, Brett D. Thombs

**Affiliations:** 1 Lady Davis Institute for Medical Research, Jewish General Hospital, 3755 Cote Ste-Catherine, Pavilion H4.83, Montréal, QC H3T 1E2 Canada; 2 Department of Psychiatry, McGill University, Montréal, QC Canada; 3 Schulich Library of Physical Sciences, Life Sciences, and Engineering, McGill University, Montréal, QC Canada; 4 Department of Psychology, McGill University, Montréal, QC Canada; 5 Department of Biostatistics and Health Informatics, King’s College London, London, UK; 6 Centre for Prognosis Research, School of Medicine, Keele University, Staffordshire, UK; 7 Centre for Addiction and Mental Health, Toronto, ON Canada; 8 Dalla Lana School of Public Health, University of Toronto, Toronto, ON Canada; 9 Li Ka Shing Knowledge Institute, Unity Health Toronto, Toronto, ON Canada; 10 School of Epidemiology and Public Health, University of Ottawa, Ottawa, ON Canada; 11 Correctional Service of Canada, Ottawa, ON Canada; 12 Department of Psychiatry, University of Toronto, Toronto, ON Canada; 13 McGill Group for Suicide Studies, Douglas Mental Health University Institute, McGill University, Montréal, QC Canada; 14 Department of Epidemiology, Biostatistics and Occupational Health, McGill University, Montréal, QC Canada; 15 Department of Medicine, McGill University, Montréal, QC Canada; 16 Respiratory Epidemiology and Clinical Research Unit, McGill University Health Centre, Montréal, QC Canada; 17 Department of Educational and Counselling Psychology, McGill University, Montréal, QC Canada; 18 Biomedical Ethics Unit, McGill University, Montréal, QC Canada

**Keywords:** Psychology, Health policy, Diseases, Psychiatric disorders

## Abstract

Women and gender-diverse individuals have faced disproportionate socioeconomic burden during COVID-19. There have been reports of greater negative mental health changes compared to men based on cross-sectional research that has not accounted for pre-COVID-19 differences. We compared mental health changes from pre-COVID-19 to during COVID-19 by sex or gender. MEDLINE (Ovid), PsycINFO (Ovid), CINAHL (EBSCO), EMBASE (Ovid), Web of Science Core Collection: Citation Indexes, China National Knowledge Infrastructure, Wanfang, medRxiv (preprints), and Open Science Framework Preprints (preprint server aggregator) were searched to August 30, 2021. Eligible studies included mental health symptom change data by sex or gender. 12 studies (10 unique cohorts) were included, all of which reported dichotomized sex or gender data. 9 cohorts reported results from March to June 2020, and 2 of these also reported on September or November to December 2020. One cohort included data pre-November 2020 data but did not provide dates. Continuous symptom change differences were not statistically significant for depression (standardized mean difference [SMD] = 0.12, 95% CI -0.09–0.33; 4 studies, 4,475 participants; I^2^ = 69.0%) and stress (SMD = − 0.10, 95% CI -0.21–0.01; 4 studies, 1,533 participants; I^2^ = 0.0%), but anxiety (SMD = 0.15, 95% CI 0.07–0.22; 4 studies, 4,344 participants; I^2^ = 3.0%) and general mental health (SMD = 0.15, 95% CI 0.12–0.18; 3 studies, 15,692 participants; I^2^ = 0.0%) worsened more among females/women than males/men. There were no significant differences in changes in proportions above cut-offs: anxiety (difference = − 0.05, 95% CI − 0.20–0.11; 1 study, 217 participants), depression (difference = 0.12, 95% CI -0.03–0.28; 1 study, 217 participants), general mental health (difference = − 0.03, 95% CI − 0.09–0.04; 3 studies, 18,985 participants; I^2^ = 94.0%), stress (difference = 0.04, 95% CI − 0.10–0.17; 1 study, 217 participants). Mental health outcomes did not differ or were worse by small amounts among women than men during early COVID-19.

## Introduction

In just over two years, the COVID-19 pandemic has caused over 6 million deaths and disrupted social and economic activities across the globe^1,2^. There has been great concern about population-wide mental health ramifications. Evidence from syntheses of longitudinal studies that have compared pre-COVID-19 symptoms to symptoms among the same study sample during the pandemic, however, has suggested that changes, if present, have been, surprisingly, generally small (e.g., standardized mean difference pre-COVID-19 to during COVID-19 = 0.10 to 0.20)^3–5^.

Small aggregate differences, however, may not reflect important differences in vulnerable populations. By sex, males infected with COVID-19 are at greater risk of intensive care admission and death than females^6,7^, but, by gender, socioeconomic burden has disproportionately impacted women^8–15^. Economically, most single parents are women, and women earn less, are more likely to live in poverty, and hold less secure jobs than men, which heightens vulnerability^8,11–14^. Women are overrepresented in health care jobs, which involves infection risk^8–13^ and provide most childcare and family elder care^8,11–13^. Intimate partner violence has increased with the majority directed towards women^8,10–13,15^. In addition to women, sex and gender minority individuals may face heightened socioeconomic challenges during COVID-19^16,17^.

Many of the socioeconomic implications of the pandemic that disproportionately affected women are known to be associated with worse mental health, and there is concern that increased burden during COVID-19 on women and gender minorities may have translated into worse mental health outcomes for these groups^18–20^. Some researchers and prominent news media stories have reported that COVID-19 mental health effects have been greater for women than men^21–31^. These reports, however, have been cross-sectional studies that evaluated proportions of participants above cut-offs on self-report measures without consideration of pre-COVID-19 differences, even though mental health disorders and symptoms were more common among women prior to the pandemic^32–36^. No evidence syntheses have directly compared data on symptom changes by sex or gender from pre-COVID-19 to during the pandemic.

Evidence from longitudinal cohorts that compare mental health symptoms pre-COVID-19 to during COVID-19 is needed to determine if there are gender differences. We are conducting a series of living systematic reviews on COVID-19 mental health^5,37,38^, including mental health changes^5^. The objective of this study was to compare mental health changes by sex or gender. This study goes beyond analyses presented in our main living systematic review, which reports on symptom levels and changes in many different population groups by conducting direct comparisons in the subset of studies that provide data on mental health changes by sex or gender.

## Methods

Our series of living systematic reviews was registered in PROSPERO (CRD42020179703) and a protocol was posted to the Open Science Framework prior to initiating searches (https://osf.io/96csg/). The present study is a sub-study of our main mental health changes review^5^. Results are reported per the PRISMA statement^39^.

### Study eligibility

For our main symptom changes review, studies on any population were included if they compared mental health outcomes assessed between January 1, 2018 and December 31, 2019, when China first reported COVID-19 to the World Health Organization^40^, to outcomes collected January 1, 2020 or later. We only included pre-COVID-19 data collected in the two years prior to COVID-19 to reduce comparisons of COVID-19 results with those collected during different developmental life stages. Compared samples had to include at least 90% of the same participants pre-COVID-19 and during COVID-19 or use statistical methods to account for missing follow-up data. Studies with < 100 participants were excluded for feasibility and due to their limited relative value. For the present analysis, studies had to report mental health outcomes separately by sex (assignment based on external genitalia, usually at birth; e.g., female, male, intersex) or gender (socially constructed characteristics of roles and behaviours; e.g., woman, man, trans woman, trans man, non-binary)^41^.

### Search strategy

MEDLINE (Ovid), PsycINFO (Ovid), CINAHL (EBSCO), EMBASE (Ovid), Web of Science Core Collection: Citation Indexes, China National Knowledge Infrastructure, Wanfang, medRxiv (preprints), and Open Science Framework Preprints (preprint server aggregator) were searched using a strategy designed by an experienced health science librarian. The China National Knowledge Infrastructure and Wanfang databases were searched using Chinese terms based on our English-language strategy. The rapid project launch did not allow for formal peer review, but COVID-19 terms were developed in collaboration with other librarians working on the topic. See Supplementary material 1 for search strategies. The initial search was conducted from December 31, 2019 to April 13, 2020 with automated daily updates. We converted to weekly updates on December 28, 2020 to increase processing efficiency.

### Selection of eligible studies

Search results were uploaded into DistillerSR (Evidence Partners, Ottawa, Canada). Duplicate references were removed. Then two reviewers independently evaluated titles and abstracts in random order; if either reviewer believed a study was potentially eligible, it underwent full-text review by two independent reviewers. Discrepancies at the full-text level were resolved by consensus, with a third reviewer consulted if necessary. An inclusion and exclusion coding guide was developed, and team members were trained over several sessions. See Supplementary material 2.

### Data extraction

For each eligible study, data were extracted in DistillerSR by a single reviewer using a pre-specified form with validation by a second reviewer. Reviewers extracted (1) publication characteristics (e.g., first author, year, journal); (2) population characteristics and demographics, including eligibility criteria, recruitment method, number of participants, assessment timing, age; (3) mental health outcomes which included symptoms of anxiety, symptoms of depression, general mental health, and stress; (4) if studies reported outcomes by sex or gender or used these terms inconsistently (e.g., described using gender but reported results for females and males, which are sex terms); and (5) if sex or gender were treated as binary or categorical.

Adequacy of study methods and reporting was assessed using an adapted version of the Joanna Briggs Institute Checklist for Prevalence Studies, which assesses appropriateness of the sampling frame for the target population, appropriateness of recruiting methods, sample size, description of setting and participants, participation or response rate, outcome assessment methods, standardization of assessments across participants, appropriateness of statistical analyses, and follow-up^42^. Each of the 9 items was coded as “yes” for meeting adequacy criteria, "no” for not meeting criteria, or “unclear” if incomplete reporting did not allow a judgment to be made. See Supplementary material 3.

For all data extraction, including adequacy of study methods and reporting, discrepancies were resolved between reviewers with a third reviewer consulted if necessary.

### Statistical analyses

For continuous outcomes, separately for each sex or gender group, we extracted a standardized mean difference (SMD) effect size with 95% confidence intervals (CIs) for change from pre-COVID-19 to COVID-19. If not provided, we extracted pre-COVID-19 and COVID-19 means and standard deviations (SDs) for each group, calculated raw change scores (SD), and calculated SMD for change using Hedges’ g for each group^43^, as described by Borenstein et al.^44^. Raw change scores were presented in scale units and direction, whereas SMD change scores were presented as positive when mental health worsened from pre-COVID-19 to COVID-19 and negative when it improved. We then calculated a Hedges’ g difference in change between sex or gender groups with 95% CI. Positive numbers represented greater negative change in females or women compared to males or men.

For studies that reported proportions of participants above a scale cut-off, for pre-COVID-19 and COVID-19 proportions, if not provided, we calculated a 95% CI using Agresti and Coull’s approximate method for binomial proportions^45^. We then extracted or calculated the proportion change in participants above the cut-off, along with 95% CI, for each sex or gender group. Proportion changes were presented as positive when mental health worsened from pre-COVID-19 to COVID-19 and negative when it improved. If 95% CIs were not reported, we generated them using Newcombe’s method for differences between binomial proportions based on paired data^46^. To do this, which requires the number of cases at both assessments, which is not typically available, we assumed that 50% of pre-COVID-19 cases continued to be cases during COVID-19 and confirmed that results did not differ substantively if we used values from 30 to 70% (all 95% CI end points within 0.02; see Supplementary Table S1). Finally, we calculated a difference of the proportion change between sex or gender groups with 95% CI^47^. Positive numbers reflected greater negative change in females or women compared to males or men.

Meta-analyses were done to synthesize differences between sex or gender groups in SMD change for continuous outcomes and in proportion change for dichotomous outcomes via restricted maximum-likelihood random-effects meta-analysis. Heterogeneity was assessed with the I^2^ statistic. Meta-analysis was performed in R (R version 3.6.3, RStudio Version 1.2.5042), using the metacont and metagen functions in the meta package^48^. Forest plots were generated using the forest function in meta. Positive values indicated more relatively worse changes in mental health for females or women compared to males or men.

## Results

### Search results and selection of eligible studies

As of August 30, 2021, there were 64,496 unique references identified and screened for potential eligibility, of which 63,534 were excluded after title and abstract review and 741 after full-text review. Of 221 remaining articles, 209 were excluded, leaving 12 included studies that reported data from 10 cohorts. Supplementary Fig. S1 shows the flow of article review and reasons for exclusion.

### Characteristics of included studies

Four publications^49–52^ reported on 2 large, national, probability-based samples from the United Kingdom (*N* = 10,918 to 15,376)^49,50^ and the Netherlands (*N* = 3,983 to 4,064),^51,52^ and one publication^53^ reported on a community sample from Spain (*N* = 102). Two studies^54,55^ assessed young adults; one reported on a sample of twins from the United Kingdom (*N* = 3,563 to 3,694 depending on outcome)^54^ and another on a sample from Switzerland (*N* = 786)^55^. One study assessed adolescents from Australia (*N* = 248)^56^, and 3 studies^57–59^ assessed undergraduate students from China (*N* = 4,085 to 4,341)^57^, India (*N* = 217)^58^, and the United Kingdom (*N* = 214)^59^. One study^60^ assessed patients with systemic lupus erythematosus (*N* = 316). Four studies assessed anxiety symptoms^54,56,58,60^, 4 depression symptoms^54,56,58,60^, 7 (5 cohorts) general mental health^49–53,57,59^, and 4 stress^55,58–60^. Table 1 shows study characteristics. All studies compared women and men or females and males; none included other sex or gender groups. Use of sex and gender terms, however, was inconsistent in 5 of 12 included studies^50,54,56,58,59^ (e.g., described assessing gender but reporting results for “females” and “males”). Results during COVID-19 were assessed between March and June 2020 for 9 cohorts^49–51,53–56,58,59^. Two cohorts also reported results from September 2020^50^ and November to December 2020^52^. One cohort did not report data collection dates but was identified in a search on November 9, 2020^57^.

**Table 1:** Characteristics of included studies (*N* = 12).

First author	Outcome domains				Description of study design and participants	Country	Pre- and post-COVID-19 dates of data collection	*N* (%) females or women (F/W) and males or men (M/M)	Mean (SD) participant age or age range (%)	Use of sex or gender
	Anxiety symptoms	Depression symptoms	General mental health	Stress						
Dong^57^			SCL-90-R		First-year undergraduate students from a single university recruited online who completed two assessments (one prior to COVID-19 and one during)	China	09/2019 pre-11/2020^a^	F/W: 3162–3,277 (75.5–77.4) M/M: 923–1064 (22.6–24.5)	19 (1)	Gender
Lim^60^	PROMIS Anxiety	PROMIS Depression		PSS	Patients with systemic lupus erythematosus from the Georgians Organized Against Lupus cohort who complete annual surveys	United States	NR/2018–2019 03–04/2020	F/W: 295 (93.4) M/M: 21 (6.6)	47 (13)	Sex
Magson^56^	SCAS-C	SMFQ-C			Adolescents aged 13–16 years who complete annual assessments as part of an ongoing longitudinal cohort that began 4 years prior	Australia	NR/2019 05/2020	Girls: 126 (50.8) Boys: 122 (49.2)	14 (1)	Inconsistent
Megías-Robles^53^			PANAS-NA		Community sample aged 19–67 years who completed two assessments (one prior to COVID-19 and one during)	Spain	11/2019 04/2020	F/W: 67 (65.7) M/M: 35 (34.3)	30 (13)	Gender
Pierce^49^ Daly^50^			GHQ-12		National probability-based sample of adults aged ≥ 18 years who are part of the United Kingdom Household Longitudinal Study; an ongoing longitudinal study which has been collecting data continuously since 2009	United Kingdom	Pre-COVID-19 waves^b^	F/W: 7181^c^ (46.7) M/M: 8195^c^ (53.3)	18–34 (11.5)^d^ 35–49 (22.4)^d^ 50–64 (34.5)^d^ 65 + (31.6)^d^	Gender Inconsistent
							04–09/2020	F/W: 6380 (58.4) M/M: 4538 (41.6)		
Rimfeld^54^	GAD-7	SMFQ			Adult twins born between 1994–1996 who, at age 18 months, were enrolled in the Twins Early Development Study; a longitudinal study which has collected over 14 waves of assessment in 20 years of data collection	United Kingdom	NR/2018 04–05/2020	F/W: 2513–2578^e^ (69.8–70.5) M/M: 1050–1116^e^ (29.5–30.2)	24–26 (100.0)	Inconsistent
Saraswathi^58^	DASS-21 Anxiety	DASS-21 Depression		DASS-21 Stress	Convenience sample of undergraduate university medical students who completed two assessments (one prior to COVID-19 and one during)	India	12/2019 06/2020	F/W: 139 (64.1) M/M: 78 (35.9)	20 (2)	Inconsistent
Savage^59^			WEMWBS	PSS	Undergraduate students from a single university recruited by email invitation and completed three or more assessments as part of the Student Health Study, a longitudinal cohort study	United Kingdom	10/2019 04/2020	F/W: 154 (72.0) M/M: 60 (28.0)	18–21 (64.5) 22–25 (22.0) 26–35 (7.5) 35 + (6.1)	Inconsistent
Shanahan^55^				PSS	Young adults who completed 9 assessments since 2004 as part of the Zurich Project on the Social Development from Childhood to Adulthood, a longitudinal cohort study	Switzerland	NR/2018 04/2020	F/W: 378 (48.1) M/M: 408 (51.9)	22 (0)	Sex
van der Velden^51^ van der Velden^52^			MHI-5		National probability-based sample of adults aged ≥ 18 years who were enrolled and completed assessments for the Longitudinal Internet Studies for the Social Sciences since 2007	The Netherlands	03/2019 11–12/2019 03/2020 11–12/2020	F/W: 2020 (50.7) M/M: 1963 (49.3) F/W: 2062 (50.7) M/M: 2002 (49.3)	18–34 (24.9)^f^ 35–49 (22.9)^f^ 50–64 (26.1)^f^ 65 + (26.1)^f^	Gender Sex

*DASS-21 Anxiety =* Depression, Anxiety, and Stress Scale—Anxiety subscale; *DASS-21 Depression* = Depression, Anxiety, and Stress Scale—Depression subscale; *DASS-21 Stress =* Depression, Anxiety, and Stress Scale—Stress subscale, *GAD-7* Generalized Anxiety Disorder-7; *GHQ-12 =* General Health Questionnaire-12; *MHI-5 =* Mental Health Index-5; *PANAS-NA* = Positive and Negative Affect Schedule—Negative affect subscale; *PROMIS Anxiety* = Patient-Reported Outcomes Measurement Information System—Anxiety subscale; *PROMIS Depression =* Patient-Reported Outcomes Measurement Information System—Depression subscale; *PSS =* Perceived Stress Scale; *SCAS-C* = Spence Children's Anxiety Scale—Child; *SCL-90-R =* Symptom Check List-90 Revised; *SMFQ =* Short Mood and Feelings Questionnaire; *SMFQ-C =* Short Mood and Feelings Questionnaire—Child Version; *WEMWBS* = Warwick Edinburgh Mental Wellbeing Scale.

^a^Authors did not provide dates of data collection. The article was identified in a November 9, 2020 search. ^b^Analyses compared COVID-19 symptom levels to preceding trends across multiple assessments. ^c^Number included in fixed effects regression analysis from where the majority of data were extracted. ^d^Age groups reported for Daly^48^; for Pierce,^49^ 16–24 = 8.8%, 25–34 = 11.2%, 35–44 = 16.0%, 45–54 = 20.1%, 55–69 = 28.9%, 70 + = 15.1%. ^e^Number of participants differed by outcome. ^f^Based on van der Velden.^51^.

### Adequacy of study methods and reporting

Two studies (1 cohort)^51,52^ were rated as “yes” for adequacy for all items. Other studies were rated “no” for 1–3 items (plus 0–3 unclear ratings)^50,53,55–59^ or “no” on none but “unclear” on 2–4 items^49,54,60^. There were 6 studies^53,55–59^ rated “no” or “unclear” for appropriate sampling frame (50.0%), 8 “no” or “unclear” for adequate response rate and coverage (66.7%)^49,50,53–56,59,60^, and 7 “no” or “unclear” for follow-up response rate and management (58.3%)^49,50,53,54,56,59,60^. See Supplementary Table S2 for results for all studies.

### Mental health symptom changes

**Figure 1:**
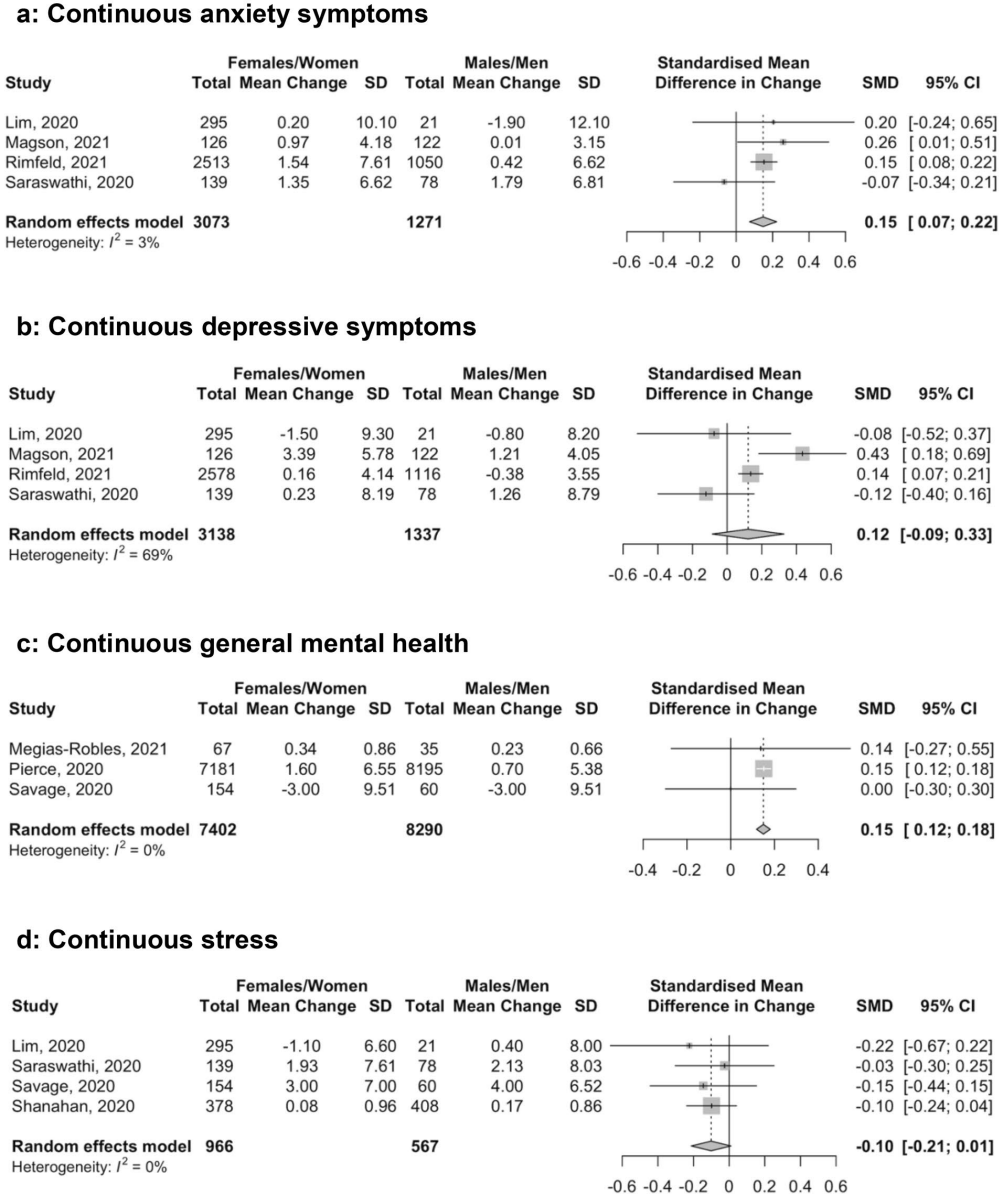
Forest plots of standardized mean difference of the difference in change in continuous anxiety symptom scores (**a**), depression symptom scores (**b**), general mental health scores (**c**), and stress scores (**d**) between females or women and males or men. Positive numbers indicate greater negative change in mental health in females or women compared to males or men.

**Figure 2:**
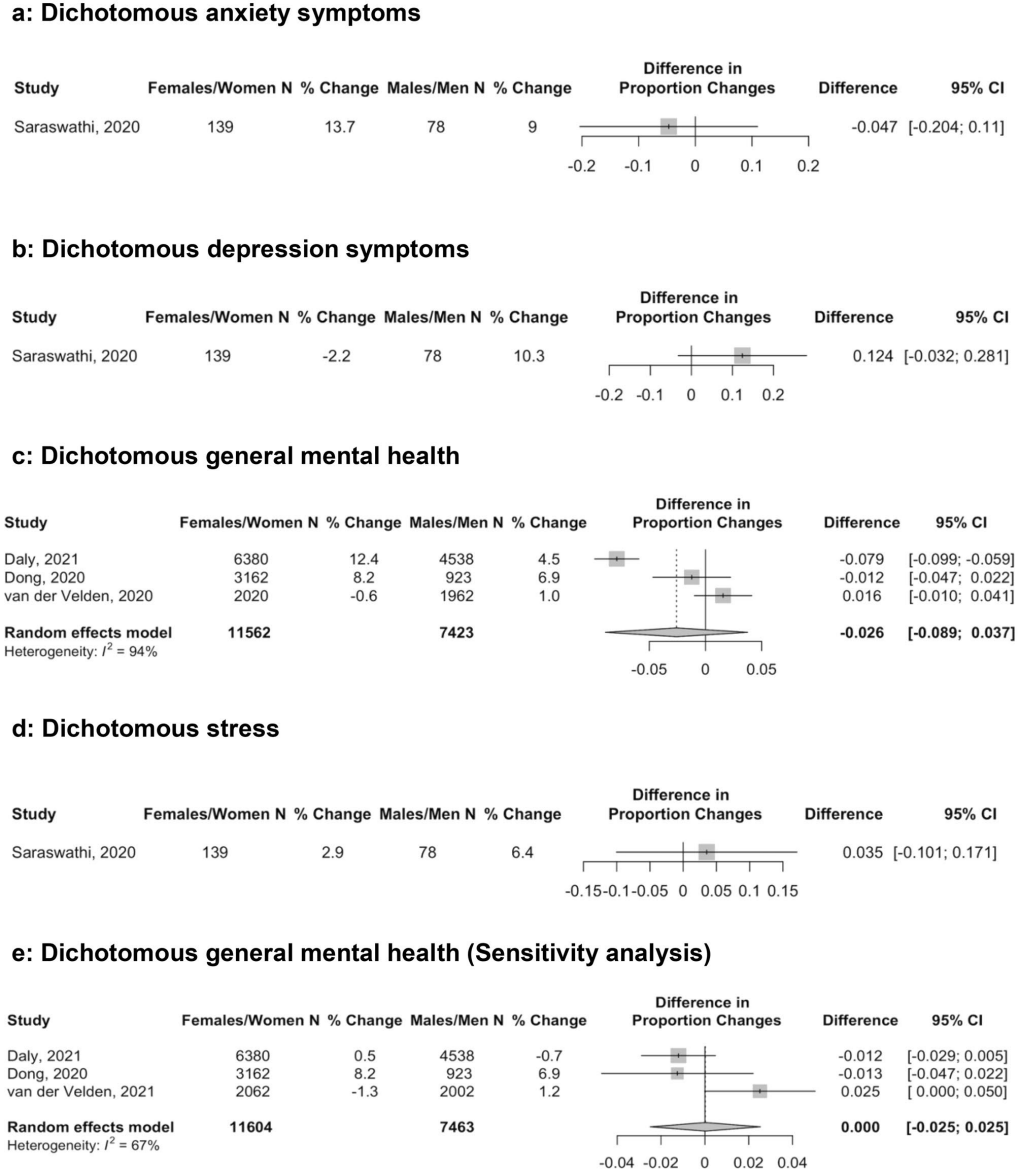
Forest plots of standardized mean difference of the difference in change in proportion above a cut-off for anxiety (**a**), depression (**b**), general mental health (**c**), and stress (**d**) between females or women and males or men. Positive numbers indicate greater negative change in mental health in females or women compared to males or men. (**c**) reflects dichotomous COVID-19 mental health measured in early 2020, whereas (**e**) reflects measurements from late 2020 for Daly^50^ and van der Velden^52^.

There was a total of 15 comparisons of continuous score changes and 6 of proportion changes; in 15 out of 21 comparisons, females or women had worse mental health pre-COVID-19. Mental health scores and symptom changes for all outcome domains are reported separately by sex or gender groups in Table 2. Differences in continuous and dichotomous changes by sex or gender are shown in Figs. 1 and 2. Estimates of difference in change by sex or gender were close to zero and not statistically significant for anxiety symptoms with dichotomous outcomes (Fig. 2a; proportion change difference = − 0.05, 95% CI − 0.20 to 0.11; *N* = 1 study^58^, 217 participants), depression symptoms with continuous (Fig. 1b; SMD change difference = 0.12, 95% CI − 0.09 to 0.33; *N* = 4 studies^54,56,58,60^, 4,475 participants; I^2^ = 69.0%) and dichotomous outcomes (Fig. 2b; proportion change difference = 0.12, 95% CI -0.03 to 0.28; *N* = 1 study^58^, 217 participants), general mental health dichotomous outcomes (Fig. 2c [all results from early 2020]; proportion change difference = − 0.03, 95% CI − 0.09 to 0.04; *N* = 3 studies^50,51,57^, 18,985 participants; I^2^ = 94.0%), and stress with continuous (Fig. 1d; SMD change difference = − 0.10, 95% CI − 0.21 to 0.01; *N* = 4 studies^55,58–60^, 1,533 participants; I^2^ = 0.0%) and dichotomous outcomes (Fig. 2d; proportion change difference = 0.04, 95% CI − 0.10 to 0.17; *N* = 1 study^58^, 217 participants). Of the 4 studies^50–52,57^ that reported dichotomous general mental health, 2 studies^50,52^ also reported outcomes from late 2020; when those results were used, the null finding did not change (Fig. 2e; proportion change difference = 0.00, 95% CI -0.03 to 0.03; *N* = 3 studies^50,52,57^ 19,067 participants; I^2^ = 67.0%).

**Table 2:** Outcomes from included studies by sex or gender.

First author	Pre- and post-COVID-19 data collection	Sex or gender	*N*	Continuous outcome measure	Pre-COVID-19 mean (SD)	Post-COVID-19 mean (SD)	Mean (SD) change		Hedges’ g standardized mean difference (95% CI)	Dichotomous outcome measure	% Pre-COVID-19 (95% CI)	% Post-COVID-19 (95% CI)	% Change (95%CI)
**Anxiety symptoms**													
Lim^60^	NR/2018–2019 03–04/2020	Females/Women	295	PROMIS Anxiety	50.20 (11.50)	50.40 (11.10)	0.20 (10.10)		0.02 (− 0.14, 0.18)	––	––	––	––
		Males/Men	21		50.50 (9.90)	48.60 (11.00)	− 1.90 (12.10)		− 0.17 (− 0.80, 0.45)				
Magson^56^	NR/2019 05/2020	Females/Women	126	SCAS-C	5.55 (4.05)	6.52 (4.31)	0.97 (4.18)		0.23 (− 0.02, 0.48)	––	––	––	––
		Males/Men	122		3.63 (3.13)	3.64 (3.16)	0.01 (3.15)		0.00 (− 0.25, 0.26)				
Rimfeld^54^	NR/2018 04–05/2020	Females/Women	2,513	GAD-7	8.15 (7.53)	9.69 (7.69)	1.54 (7.61)		0.20 (0.15, 0.26)	––	––	––	––
		Males/Men	1,050		5.88 (6.66)	6.30 (6.58)	0.42 (6.62)		0.06 (− 0.02, 0.15)				
Saraswathi^58^	12/2019 06/2020	Females/Women	139	DASS-21 Anxiety	4.59 (6.29)	5.94 (6.93)	1.35 (6.62)		0.20 (− 0.03, 0.44)	DASS-21 Anxiety > 7	18.7 (13.1, 26.0)	32.4 (25.2, 40.5)	13.7 (4.4, 22.7)
		Males/Men	78		4.62 (6.04)	6.41 (7.50)	1.79 (6.81)		0.26 (− 0.06, 0.58)		25.6 (17.3, 36.3)	34.6 (25.0, 45.7)	9.0 (-4.0, 21.5)
**Depression symptoms**													
Lim^60^	NR/2018–2019 03–04/2020	Females/Women	295	PROMIS Depression	50.80 (10.70)	49.30 (9.80)	− 1.50 (9.30)		− 0.15 (− 0.31, 0.02)	––	––	––	––
		Males/Men	21		50.70 (8.60)	49.90 (9.70)	− 0.80 (8.20)		− 0.08 (− 0.70, 0.54)				
Magson^56^	NR/2019 05/2020	Females/Women	126	SMFQ-C	4.77 (5.00)	8.16 (6.46)	3.39 (5.78)		0.58 (0.33, 0.84)	––	––	––	––
		Males/Men	122		2.81 (3.18)	4.02 (4.76)	1.21 (4.05)		0.30 (0.04, 0.55)				
Rimfeld^54^	NR/2018 04–05/2020	Females/Women	2,578	SMFQ	4.65 (4.20)	4.81 (4.07)	0.16 (4.14)		0.04 (− 0.02, 0.09)	––	––	––	––
		Males/Men	1,116		3.71 (3.70)	3.33 (3.40)	− 0.38 (3.55)		− 0.11 (− 0.19, − 0.02)				
Saraswathi^58^	12/2019 06/2020	Females/Women	139	DASS-21 Depression	7.71 (7.57)	7.94 (8.77)	0.23 (8.19)		0.03 (− 0.21, 0.26)	DASS-21 Depression > 9	36.7 (29.1, 45.0)	34.5 (27.1, 42.8)	-2.2 (-11.7, 7.4)
		Males/Men	78		7.28 (8.40)	8.54 (9.17)	1.26 (8.79)		0.14 (− 0.17, 0.45)		26.9 (18.3, 37.7)	37.2 (27.3, 48.3)	10.3 (-2.9, 22.9)
**General mental health**													
Dong^57^	09/2019 NR/2020	Females/Women	3,162–3,277	––	––	––	––		––	SCL-90-R ≥ 160	19.7 (18.4, 21.1)	27.9 (26.4, 29.5)	8.2 (6.3, 10.0)
		Males/Men	923–1,064								14.3 (12.3, 16.5)	21.2 (18.7, 24.0)	6.9 (4.0, 9.9)
Megías-Robles^53^	11/2019 04/2020	Females/Women	67	PANAS-NA	1.93 (0.65)	2.28 (0.79)	0.34 (0.86)		0.46 (0.12, 0.81)	––	––	––	––
		Males/Men	35		1.88 (0.67)	2.11 (0.66)	0.23 (0.66)		0.34 (− 0.14, 0.82)				
Pierce^49^ Daly^50^	Pre-COVID-19 Waves 04/2020 09/2020	Females/Women	7,181^22,b^ 6,380	GHQ-12	12.00 (5.91)	13.60 (7.14)	1.60 (6.55)^c^ 0.88 (NR)^d^		0.24 (0.21, 0.28) 0.13 (0.10, 0.17)	GHQ-12 ≥ 4	24.5 (22.5, 26.4)^e^ 24.5 (22.5, 26.4)^e^	36.8 (34.8, 38.9)^e^ 25.0 (23.3, 26.8)^e^	12.4 (9.9, 14.9)^e^ 0.5 (− 1.8, 2.9)^e^
		Males/Men	8,195^22,b^ 4,538		10.80 (4.99)	11.50 (5.75)	0.70 (5.38)^c^ 0.03 (NR)^d^		0.13 (0.10, 0.16) 0.01 (− 0.03, 0.04)		16.7 (14.6, 18.7)^e^ 16.7 (14.6, 18.7)^e^	21.1 (19.0, 23.3)^e^ 16.0 (14.0, 17.9)^e^	4.5 (2.0, 7.0)^e^ − 0.7 (− 2.9, 1.5)^e^
Savage^59^	10/2019 04/2020	Females/Women	154	WEMWBS^f^	43.00 (9.00)	40.00 (10.00)	− 3.00 (9.51)		0.31 (0.09, 0.54)	––	––	––	––
		Males/Men	60		47.00 (9.00)	44.00 (10.00)	− 3.00 (9.51)		0.31 (− 0.05, 0.67)				
van der Velden^51^ van der Velden^52^	03/2019 11–12/2019 03/2020 11–12/2020	Females/Women	2,020 2,062	––	––	––	––		––	MHI-5 ≤ 59	18.9 (17.3, 20.7) 19.1 (17.4, 20.8)	18.3 (16.7, 20.1) 17.8 (16.2, 19.5)	− 0.6 (− 2.5, 1.3) − 1.3 (− 3.1, 0.6)
		Males/Mmen	1,962–1,963 2,002								14.6 (13.1, 16.3) 14.7 (13.2, 16.3)	15.6 (14.1, 17.3) 15.9 (14.4, 17.6)	1.0 (− 0.8, 2.7) 1.2 (− 0.5, 3.0)
**Stress**													
Lim^60^	NR/2018-2019 03-04/2020	Females/Women	295	PSS	17.50 (8.00)	16.40 (8.20)	− 1.10 (6.60)		− 0.14 (− 0.30, 0.03)	––	––	––	––
		Males/Men	21		17.70 (6.60)	18.10 (6.80)	0.40 (8.00)		0.06 (− 0.56, 0.68)				
Saraswathi^58^	12/2019 06/2020	Females/Women	139	DASS-21 Stress	6.95 (7.22)	8.88 (7.99)	1.93 (7.61)		0.25 (0.02, 0.49)	DASS-21 Stress > 14	19.4 (13.7, 26.8)	22.3 (16.2, 29.9)	2.9 (− 4.9, 10.6)
		Males/Men	78		7.95 (7.54)	10.08 (8.50)	2.13 (8.03)		0.26 (− 0.05, 0.58)		23.1 (15.1, 33.6)	29.5 (20.5, 40.4)	6.4 (− 5.6, 18.2)
Savage^59^	10/2019 04/2020	Females/Women	154	PSS	21.00 (7.00)	24.00 (7.00)	3.00 (7.00)	0.43 (0.20, 0.65)		––	––	––	––
		Males/Men	60		17.00 (6.00)	21.00 (7.00)	4.00 (6.52)	0.61 (0.24, 0.97)					
Shanahan^55^	NR/2018 04/2020	Females/Women	378	PSS	3.02 (0.98)	3.10 (0.94)	0.08 (0.96)	0.08 (− 0.06, 0.23)		––	––	––	––
		Males/Men	408		2.57 (0.86)	2.74 (0.86)	0.17 (0.86)	0.20 (0.06, 0.34)					

*DASS-21 Anxiety =* Depression, Anxiety, and Stress Scale—Anxiety subscale; *DASS-21 Depression =* Depression, Anxiety, and Stress Scale—Depression subscale; *DASS-21 Stress* = Depression, Anxiety, and Stress Scale—Stress subscale; *GAD-7 =* Generalized Anxiety Disorder-7; *GHQ-12* = General Health Questionnaire-12; *MHI-5 =* Mental Health Index-5; *PANAS-NA =* Positive and Negative Affect Schedule—Negative affect subscale; *PROMIS Anxiety =* Patient-Reported Outcomes Measurement Information System—Anxiety subscale; *PROMIS Depression =* Patient-Reported Outcomes Measurement Information System—Depression subscale; *PSS* = Perceived Stress Scale; *SCAS-C* = Spence Children's Anxiety Scale—Child; *SCL-90-R =* Symptom Check List-90 Revised; *SMFQ =* Short Mood and Feelings Questionnaire; *SMFQ-C =* Short Mood and Feelings Questionnaire—Child Version; *WEMWBS =* Warwick Edinburgh Mental Wellbeing Scale.

^a^Positive Hedge’s g sizes and increases in proportions above a threshold indicate worse mental health in COVID-19 compared to pre-COVID-19. Effects for measures where high scores = positive outcomes were reversed to reflect this. ^b^ Number included in fixed effects regression analysis from where majority of data were extracted. ^c^Based on difference between 2020 and 2019 outcomes. ^d^Based on estimate from fixed effects regression model that estimates within-person change accounting for pre-COVID-19 trends.^e^Included proportion outcomes from Daly^50^ since they reported for two time points. ^f^ Higher scale scores reflect better mental health; thus, direction of effect sizes reversed.

**Figure 3:**
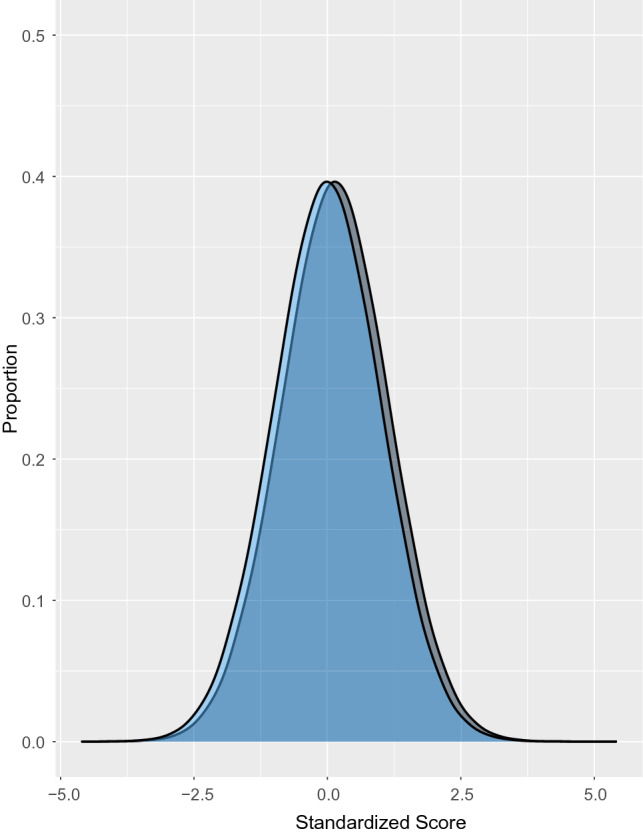
Illustration of the magnitude of change for SMD = 0.15 assuming a normal distribution. The hypothetical blue distribution represents pre-COVID-19 scores, and the grey distribution represents post-COVID-19 scores with a mean symptom increase of SMD = 0.15.

Anxiety, measured continuously, worsened significantly more for females or women than for males or men during COVID-19 (Fig. 1a; SMD change difference = 0.15, 95% CI 0.07 to 0.22; *N* = 4 studies^54,56,58,60^, 4,344 participants; I^2^ = 3.0%). General mental health, measured continuously, also worsened more for females or women than for males or men in early COVID-19 (Fig. 1c; SMD difference in change = 0.15, 95% CI 0.12 to 0.18; *N* = 3 studies^49,53,59^, 15,692 participants; I^2^ = 0.0%). This was predominantly based on a large population-based study from the United Kingdom^49^. That study did not report results from fall 2020 for continuous outcomes, but as shown in Table 2 and Figs. 2c and e, the difference in change between females or women and males or men decreased between early and late 2020 for dichotomous outcomes in the same cohort^50^. The magnitude of both statistically significant differences was small (see Fig. 3).

## Discussion

The COVID-19 pandemic has affected women and gender minorities disproportionately^8–17^. There has been an assumption, seemingly confirmed by cross-sectional data collected during COVID-19, that overall mental health has worsened and that there have been even greater negative changes in mental health among women than for men^21–31^. We reviewed evidence from 12 studies (10 cohorts) that reported mental health changes from pre-COVID-19 to COVID-19 separately by sex or gender. We compared females or women with males or men; no studies compared gender minorities with any other group. Data were largely from March to June 2020, early in the pandemic. Syntheses of continuously measured anxiety symptoms (SMD = 0.15, 95% CI 0.07 to 0.22) and general mental health (SMD = 0.15, 95% CI 0.12 to 0.18) found that mental health worsened more for females or women than males or men, but the magnitude was small and far below thresholds that are typically considered clinically important (e.g., SMD = 0.50)^61^. None of the other 6 mental health outcomes that we examined (continuous depression symptoms and stress; dichotomous anxiety symptoms, depression symptoms, general mental health, and stress) differed by sex or gender.

Sex and gender differences in mental health disorder prevalence, symptoms, and risk factors are well-established^62–65^. Likely risk factors include gender inequities and discrimination, economic disadvantage and poverty, higher rates of interpersonal stressors, and violence^66,67^, and many of these risk factors have been exacerbated for women during COVID-19^8–15^. We did not identify any differences in mental health by sex or gender, however, that appeared to be substantive; all were 0.15 SMD or smaller, which is considered to be a small difference based on commonly used metrics (e.g., < 0.20 SMD)^68^ and below thresholds for clinical meaningfulness^61^.

Based on our findings, it is possible that despite the challenges women have faced, many have been resilient and that the mental health disaster that has been predicted by many has not occurred^69^. Overall, across populations, expected negative changes in mental health during the pandemic compared to pre-pandemic levels have not been as dramatic as might have been expected^3,70–72^. To the best of our knowledge, there have been two systematic reviews that have compared symptoms prior to COVID-19 and after the start of the pandemic. The reviews used somewhat different methods, including study inclusion and exclusion criteria, but findings were consistent. Both reported that symptom scores on measures of general mental health, depression, and anxiety were stable or had worsened by small amounts during the pandemic^4,5^. This is consistent with the only study, to the best of our knowledge, that has evaluated prevalence of mental health disorders using validated diagnostic interviews rather than symptom changes. That study, which probabilistically sampled Norwegian adults in January to early March 2020 (pre-pandemic), mid-March to May 2020, and June to July 2020, reported that the prevalence of current mental disorders, assessed using the Composite International Diagnostic Interview (version 5.0), was stable across time periods^73^. Similarly, a study on suicide in 21 countries during early COVID-19 found that observed numbers of deaths from suicide was stable or decreased from pre-pandemic to the early pandemic months in all included jurisdictions based on an interrupted time-series analysis^72^.

Our findings, as well as those from other studies that have reported that mental health implications early in the pandemic may not have been as substantial as expected depart from what has been reported in some research and by the media. Three factors may feed this discrepancy. One is the publication of many cross-sectional studies that report proportions above cut-offs on self-report measures, which are not designed for that purpose^74–78^, and assume that what are perceived as high numbers, generally, or sex differences, comparatively, must not have been present pre-COVID-19^5^. A second is the use of surveys that ask questions about well-being with COVID-19 explicitly assigned as a cause; illustrating the pitfalls of this, a study of over 2,000 young Swiss adult men found significant angst when questions were asked in this way, but no changes in validated measures of depression symptoms and stress from pre-COVID^79^. A third reason relates to news media reports that emphasize dramatic events and anecdotes without evidence that demonstrates changes^69^.

Strengths of our study include the use of rigorous systematic review methods. We searched 9 databases, including Chinese-language databases, without language restrictions and included studies that enabled the direct comparison of mental health changes by sex or gender. Our findings emphasize that we should not assume that mental health effects of COVID-19 have been much greater for females or women than for males or men during the pandemic. Indeed, across the 21 analyses we conducted, differences were consistently null or very small and no individual studies stood out as deviating from this overall finding. Nonetheless, one should be cautious about generalizing our findings to all populations and subgroups. First, included studies were conducted in 8 countries, and it is possible that there could have been differences in other countries, given that the pandemic has manifested itself differently across countries and that countries have managed the pandemic differently (e.g., length and severity of restrictions). Second, all but one of the included studies was on adults, and the findings may not be generalizable to children or adolescents. Third, there were not enough studies to attempt subgroup analyses by sociodemographic or other factors, such as professional groups, for example. Cross-sectional studies have reported that there could be differences in mental health by sex or gender that are related to sociodemographic variables (e.g., age, race or ethnicity) and professional roles (e.g., health care workers)^80,81^. Cross-sectional analyses, however, do not allow us to determine if any identified associations or differences may have been present prior to the pandemic, and if so, to what degree. Fourth, we were not able to evaluate the influence of potential risk and protective factors that may differ between sex or gender and if these might potentially explain some of the results observed. The information needed to do this was not provided in included studies. Fifth, we did not identify any studies that compared results from gender-diverse individuals to other gender groups. This highlights an important evidence gap in the literature, and indicates the need for more research on this population, especially given that several studies suggest that the mental health of this population group may have been affected negatively since pre-COVID-19^16,82,83^.

There are other limitations to consider in addition to generalizability. First, this review only included 12 studies from 10 cohorts, and many had limitations related to study sampling frames and recruitment methods, follow-up rates, and management of missing data. Second, our review only included studies with mental health outcomes early in the pandemic. This did not permit us to examine long-term trends in mental health as the pandemic progressed. It is possible that sex or gender differences absent in the early pandemic may have developed. For example, according to a United States Centers for Disease Control report on suicide-related weekly emergency department visits, the numbers for teenage females (aged 12–17 years) increased minimally in 2020, but were over 51% higher in 2021 compared to the same period in 2019, versus an increase of 4% among teenage males^84^. Analyses of overall mental health that have been reported and the results in our study are based on data from early in the pandemic, and it is not clear to what degree these findings would apply to later stages of the pandemic. Third, heterogeneity was high for some meta-analyses; it was low, however, for others, and results across 8 analyses did not differ substantively. Fourth, in calculating 95% CIs for within-group changes in proportions with the information provided in publications (pre-COVID-19 and COVID-19 group proportions), we assumed that 50% of pre-COVID-19 cases continued to be cases during COVID-19. However, the maximum difference in any end point of a 95% CIs across analyses was 0.02 when we varied our assumption from 30 to 70%.

In sum, we identified small sex- or gender-based differences for anxiety symptoms and general mental health, continuously measured, but other outcomes (continuous depression symptoms and stress; dichotomous anxiety symptoms, depression symptoms, general mental health, and stress) did not differ by sex or gender. This finding diverges from what has been reported from cross-sectional studies. These are aggregate results, though, and many individuals have certainly experienced negative mental health changes related to increased socioeconomic burden. It seems plausible, given the divergent ways that the pandemic has affected different people that many people are experiencing improved mental health, whereas large numbers of others may be experiencing worsened mental health, including new onset mental disorders among people without previous morbidity. Thus, mental health changes should continue to be monitored longitudinally in COVID-19, taking into consideration sex and gender, particularly in younger populations. Our research underlines that few studies report results by sex and gender. Sex and Gender Equity in Research (SAGER) guidelines^85^ emphasize that all studies should report results by sex and gender, even if there are not enough participants to draw sex- and gender-based conclusions. Reporting by sex and gender, even in small studies, facilitates synthesis of results across studies, which does allow conclusions to be drawn, even if primary studies do not have sufficiently large samples sizes to do this. Ongoing research in COVID-19 should include outcome reports by sex and gender. When not done, peer reviewers and editors should support authors to implement this guidance. Although we did not find aggregate sex differences and overall changes have been minimal, the pandemic has affected different individuals and groups differently. Health care providers should be alert to life changes that may be associated with vulnerability and to physical and emotional or cognitive symptoms that may reflect worsening mental health so that they can assess, if appropriate, and provide mental health care to those in need.

## Supplementary Information

Supplementary Information.

## Data Availability

All data used in the study are available in the manuscript and its tables or online at https://www.depressd.ca/covid-19-mental-health.
